# Checklist of the superfamilies Conopoidea, Diopsoidea and Nerioidea of Finland (Insecta, Diptera)

**DOI:** 10.3897/zookeys.441.7227

**Published:** 2014-09-19

**Authors:** Jere Kahanpää, Jens-Hermann Stuke

**Affiliations:** 1 Finnish Museum of Natural History, Zoology Unit, P.O. Box 17, FI-00014 University of Helsinki, Finland; 2 Roter Weg 22, 26789 Leer, Germany

**Keywords:** Checklist, Finland, Diptera, biodiversity, faunistics

## Abstract

A checklist of the Diptera superfamilies Conopoidea (Conopidae), Nerioidea (Micropezidae, Pseudopomyzidae) and Diopsoidea (Megamerinidae, Psilidae, Strongylophthalmyiidae, Tanypezidae) from Finland in presented. *Myopa vicaria* Walker, 1849 is formally recorded for the first time from the country.

## Introduction

Four fly families form the superfamily Nerioidea. Two of them, Micropezidae and Pseudopomyzidae, occur in Finland. Neriids are mostly tropical and cypselomatids (*sensu stricto*) have only been found in the Australasian and Oriental regions. The pseudopomyzids were included in Cypselosomatidae Hendel, 1931 in the previous checklist (Hackman 1980) and are still often included in Cypselosomatidae *sensu lato*. The Finnish micropezid fauna is relatively well known. One or two species of *Cnodacophora* Czerny, 1930 and *Rainieria* Rondani, 1843 could be present but are so far undetected.

Diopsoidea consists of seven to ten families (depending on the source), none particularly species-rich. Only four are found in Finland: Psilidae, Tanypezidae, Strongylophthalmyiidae and Megamerinidae. Tanypezidae and Strongylophthalmyiidae are here treated as separate families following Lonsdale (2013). Megamerinidae has been assigned to several superfamilies, most often Diopsoidea following McAlpine (1989), but also Nerioidea, Sciomyzoidea, and recently Opomyzoidea (Wiegmann et al. 2011). These families have raised no particular interest among Finnish dipterists. The genus *Chamaepsila* Hendel, 1917 in particular needs more attention. It is likely that a few Finnish species remain unrecognised in this genus.

The systematic position of the Conopidae is unclear. It has traditionally been considered basal within Schizophora. Wiegmann et al. (2011) listed Conopidae within Sciomyzoidea while others (see Skevington et al. 2010) place the family near Tephritoidea. Within the family the recent classification of Gibson and Skevington (2013) is followed. The Finnish conopid fauna is now relatively well-understood and few additional species are expected to occur in the country.

The Finnish species of these families were last listed by Hackman (1980).

**Table 1.** Number of species by family.

Family	Number of species in			Level of knowledge
	World (Pape et al. 2011)	Europe	Finland	
Conopidae	~796	83	19	good
**Nerioidea:**				
Micropezidae	573	22	6	average
Pseudopomyzidae	18	1	1	good
**Diopsoidea:**				
Psilidae	320	47	29	average
Tanypezidae	27 (Lonsdale 2013)	1	1	good
Strongylophthalmyiidae	47 (Lonsdale 2013)	2	2	good
Megamerinidae	15	1	1	good

## Checklist part 1: Conopoidea

suborder Brachycera Macquart, 1834

clade Eremoneura Lameere, 1906

clade Cyclorrhapha Brauer, 1863

infraorder Schizophora Becher, 1882

? clade Archischiza Enderlein, 1936

superfamily Conopoidea Latreille, 1802

**CONOPIDAE** Latreille, 1802

CONOPINAE Latreille, 1802

***CONOPS*** Linnaeus, 1758

**sg. *Conops*** Linnaeus, 1758

*Conops quadrifasciatus* De Geer, 1776

*Conops strigatus* Wiedemann, 1824

*Conops vesicularis* Linnaeus, 1761

***PHYSOCEPHALA*** Schiner, 1861

*Physocephala nigra* (De Geer, 1776)

ZODIONINAE Rondani, 1856

***ZODION*** Latreille, 1796

*Zodion cinereum* (Fabricius, 1794)

= *notatum* Meigen, 1804

SICINAE Zimina, 1960

***SICUS*** Scopoli, 1763

*Sicus ferrugineus* (Linnaeus, 1761)

MYOPINAE Macquart, 1834

***MYOPA*** Fabricius, 1775

*Myopa buccata* (Linnaeus, 1758)

*Myopa fasciata* Meigen, 1804

*Myopa hirsuta* Stuke & Clements, 2008

= *strandi* auct. nec Duda, 1940

*Myopa occulta* Wiedemann, 1824

= *nigrifrons* von Bonsdorff, 1866

*Myopa tessellatipennis* Motschulsky, 1859

= *polystigma* auct. nec Rondani, 1857

*Myopa testacea* (Linnaeus, 1767)

*Myopa vicaria* Walker, 1849

= *villosa* Ringdahl, 1945

= *strandi* Duda, 1940

***MYOPOTTA*** Zimina, 1969

*Myopotta pallipes* (Wiedemann, 1824)

***THECOPHORA*** Rondani, 1845

= ***Occemyia*** Robineau-Desvoidy, 1853

*Thecophora cinerascens* (Meigen, 1804)

= *pusilla* (Meigen, 1824)

= *atra* misid.

*Thecophora distincta* (Wiedemann, 1824)

= *melanopa* misid.

*Thecophora fulvipes* (Robineau-Desvoidy, 1830)

= *sundewalli* (Zetterstedt, 1844)

*Thecophora jakutica* Zimina, 1974

= *atra* misid.

DALMANNIINAE Hendel, 1916

***DALMANNIA*** Robineau-Desvoidy, 1830

*Dalmannia dorsalis* (Fabricius, 1794)

= *punctata* misid.

## Checklist part 2: Nerioidea and Diopsoidea

suborder Brachycera Macquart, 1834

clade Eremoneura Lameere, 1906

clade Cyclorrhapha Brauer, 1863

infraorder Schizophora Becher, 1882

clade Muscaria Enderlein, 1936

parvorder Acalyptratae Macquart, 1835

superfamily Nerioidea Westwood, 1840

**MICROPEZIDAE** Blanchard, 1840

CALOBATINAE Bigot, 1853

***CALOBATA*** Meigen, 1803

*Calobata petronella* (Linnaeus, 1761)

***NERIA*** Robineau-Desvoidy, 1830

*Neria cibaria* (Linnaeus, 1761)

*Neria commutata* (Czerny, 1930)

= *nigricornis* auct. nec Zetterstedt, 1838

*Neria ephippium* (Fabricius, 1794)

*Neria nigricornis* (Zetterstedt, 1838)

= *helleni* (Frey, 1918)

= *nitidicollis* (Frey, 1947)

MICROPEZINAE Blanchard, 1840

***MICROPEZA*** Meigen, 1803

*Micropeza corrigiolata* (Linnaeus, 1767)

**PSEUDOPOMYZIDAE** McAlpine, 1966

***PSEUDOPOMYZA*** Strobl, 1893

*Pseudopomyza atrimana* (Meigen, 1830)

superfamily Diopsoidea Billberg, 1820

**MEGAMERINIDAE** Hendel, 1913

***MEGAMERINA*** Rondani, 1861

= ***Lissa*** Meigen, 1826 preocc.

*Megamerina dolium* (Fabricius, 1805)

= *loxocerina* (Fallén, 1820)

**PSILIDAE** Macquart, 1835

CHYLIZINAE Rondani, 1856

***CHYLIZA*** Fallén, 1820

**sg. *Chyliza*** Fallén, 1820

*Chyliza annulipes* Macquart, 1835

= *fuscipennis* auct. nec Robineau-Desvoidy, 1830

*Chyliza leptogaster* (Panzer, 1798)

= *scutellata* (Fabricius, 1798)

*Chyliza nova* Collin, 1944

*Chyliza vittata* Meigen, 1826

PSILINAE Macquart, 1835

tribe Loxocerini Macquart, 1835

***IMANTIMYIA*** Frey, 1925

*Imantimyia albiseta* (Schrank, 1803)

= *ichneumonea* (Linnaeus, 1758) nom. dubium (see Notes)

*Imantimyia fulviventris* (Meigen, 1826)

*Imantimyia nigrifrons* (Macquart, 1835)

*Imantimyia sylvatica* (Meigen, 1826)

***LOXOCERA*** Meigen, 1803

**sg. *LOXOCERA*** Meigen, 1803

*Loxocera aristata* (Panzer, 1801)

= *ichneumonea* (Linnaeus, 1758) nom. dubium (see Notes)

tribe Psilini Macquart, 1835

***CHAMAEPSILA*** Hendel, 1917

= ***Tetrapsila*** Frey, 1925

*Chamaepsila atra* (Meigen, 1826)

*Chamaepsila bicolor* (Meigen, 1826)

= *nigromaculata* (Strobl, 1909)

*Chamaepsila buccata* (Fallén, 1826)

= *gracilis* (Meigen, 1826)

*Chamaepsila humeralis* (Zetterstedt, 1847)

*Chamaepsila limbatella* (Zetterstedt, 1847)

*Chamaepsila morio* (Zetterstedt, 1835)

*Chamaepsila nigra* (Fallén, 1820)

*Chamaepsila nigricornis* (Meigen, 1826)

*Chamaepsila nigrosetosa* Frey, 1925

*Chamaepsila obscuritarsis* (Loew, 1856)

*Chamaepsila pallida* (Fallén, 1820)

*Chamaepsila pectoralis* (Meigen, 1826)

*Chamaepsila rosae* (Fabricius, 1794)

*Chamaepsila rufa* (Meigen, 1826)

*Chamaepsila unilineata* (Zetterstedt, 1847)

***PSILA*** Meigen, 1803

**sg. *FREYOPSILA*** Shatalkin, 1986

*Psila sibirica* (Frey, 1925)

**sg. *PSILA*** Meigen, 1803

*Psila fimetaria* (Linnaeus, 1761)

*Psila merdaria* Collin, 1944

***PSILOSOMA*** Zetterstedt, 1860

*Psilosoma audouini* (Zetterstedt, 1835)

*Psilosoma lefebvrei* (Zetterstedt, 1835)

**STRONGYLOPHTHALMYIIDAE** Hendel, 1917

***STRONGYLOPHTHALMYIA*** Heller, 1902

*Strongylophthalmyia pictipes* Frey, 1935

*Strongylophthalmyia ustulata* (Zetterstedt, 1847)

**TANYPEZIDAE** Rondani, 1856

***TANYPEZA*** Fallén, 1820

*Tanypeza longimana* Fallén, 1820

## Excluded species

*Dalmannia punctata* (Fabricius, 1794) not found within present borders.

## Notes

***Chamaepsila unilineata* (Zetterstedt, 1847)** and ***Chamaepsila nigrosetosa* Frey, 1925** were synonymized with *Chamaepsila pallida* but they have recently been reinstated as valid species (Shatalkin and Merz 2010).

***Loxocera ichneumonea* (Linnaeus, 1758)**. This name has been used for *Loxocera aristata* (Panzer) and *Loxocera albiseta* (Schrank). Unfortunately no type material is known. Linné’s original description is insufficient and may in fact belong to a hoverfly (see Buck and Marshall 2006).

***Myopa vicaria* Walker, 1849**. One of the common *Myopa* species through Finland, but previously unreported due to difficulties in identification. Recorded from provinces *Ab*, *N*, *Ka*, *St*, *Ta*, *Sa*, *Kl*, *Om*, *Ok*, *Lkoc* and *Li*.
